# The effectiveness of a blended POCUS curriculum on achieving basic focused bedside transthoracic echocardiography (TTE) proficiency. A formalized pilot study

**DOI:** 10.1186/s12947-021-00268-9

**Published:** 2021-12-09

**Authors:** Jonathan Kline, Mary Golinski, Brian Selai, Jeremy Horsch, Katie Hornbaker

**Affiliations:** 1 Twin Oaks Anesthesia, Wesley Chapel, Florida USA; 2 Oakland University Nurse Anesthesia Program, Rochester Hills, MI USA

**Keywords:** Transthoracic echocardiography (TTE), TTE learning, Blended TTE platform, TTE study

## Abstract

**Objective:**

The study objective is to evaluate the effeteness of an existing educational platform blending didactic presentation and hands-on simulation for university doctoral SRNAs in the area of basic, 4 view identification and performance of transthoracic echocardiography (TTE).

**Methods:**

Following IRB approval, SRNAs were exposed to a pre test to evaluate existing skills, then they were exposed to a graphic rich, live presentation of basic 4 view TTE. The presentation was then followed by hands on simulation and performance of the 4 basic TTE views on live models.

**Results:**

Pretest scores averaged 58% and post tests scores rose to 95%. See Table 1.

**Conclusion:**

Our results support the concept that the existing blended platform is effective to train university SRNAs in basic 4 view, bedside transthoracic echocardiography.

## Background

Achieving proficiency in identifying and performing focused transthoracic echocardiography (TTE), defined as four dynamic ultrasound images of the heart, is deemed a valuable point of care ultrasound (POCUS) skill set. Numerous studies [1–10] support the hypothesis that POCUS skills are easily attainable and that varied curricular methods promote learning. Disciplines such as critical care, emergency medicine, sonographers, and residents in anesthesiology have been formally studied in terms of learning POCUS. The research reinforces that learning occurs best when didactic curriculum is coupled with a simulation hands-on type experience.

Performing POCUS at the bedside, and specifically TTE, is now considered an important component of an advanced pre-anesthesia assessment in several patient care scenarios. Current anesthesia professionals are recognizing the criticality in acquiring this skill set with the overarching goal of promoting favorable patient care outcomes related to the information known about the health status. In addition, the Council on Accreditation of Nurse Anesthesia Educational Programs (COA) has revised their educational standards for accredited graduate nurse anesthesia programs and now include POCUS. This adds further importance to establishing an efficacious platform to manage POCUS material to the nurse anesthesia education institutions.

TTE skills of basic view identification and image production offer valuable and important patient physiologic detail for certain patients who present for surgery and anesthesia. Preoperative physiologic detail and health conditions achieved by performing TTE can lead to pathology identification initiating, formalized studies, comprehensive planning and help determine the most appropriate anesthetic. However, developing POCUS programs for providers and execution of these programs are still in their infancy. Development of bedside TTE skills has yet to be characterized in both content and execution. The effectiveness of teaching TTE skills and associated educational curriculum, and the capacity to assimilate the information by entry level anesthesia providers, is not yet known. To do so requires a formalized evaluation of a TTE program, curriculum experts teaching TTE, and the SRNA participants who can be assessed for learning. While evidence exists supporting the incorporation of TTE skills effectively into medical residency training programs, none thus far have addressed the importance of adding this valuable skill to nurse anesthesia students in graduate educational programs. We therefore formally evaluated the effectiveness of an existing blended teaching program that included focused, bedside TTE concepts, basic view identification, skill attainment, and evaluated SRNA learning.

### Review of relevant literature

In 2021, Neelankavil et al compared a formalized simulation based TTE learning platform vs. a traditional non- simulation platform. Approximately 60 anesthesia residents participated in the study and those in the simulation group scored significantly higher compared to the non-simulation based training (control) group. This research also supports the incorporation of a hands- on simulation component to bedside TTE skills development [2]. A similar finding was noted by Kusunose et al (2016) after evaluating a simulation based bedside basic view TTE skills instructional platform. Results confirmed that when simulation was an integral component of the instruction, posttest scores were higher than a non- simulation group [3].

In addition, Vignon et al., evaluated learning by “non-cardiologist” ICU residents. The residents received three hours of didactic TTE curriculum followed by five hours of guided hands-on simulation at the bedside. They established that ICU residents without any prior focused TTE content and using POCUS applications can be taught and performed by novices in this arena [8]. Gibbs [9] also emphasized the importance of simulation ‘laboratory experiences’ as well as didactic curriculum for teaching sonography students. Faculty observed the student sonographers demonstrated improved hand- eye coordination after practicing in the simulation laboratory and that teaching the fundamental material to the students took less time when simulation laboratory experiences were part of the entire curriculum. The students also communicated an increase in familiarity with the procedure and confidence with scanning concepts before touching a patient or live model.

Further considering a blending teaching platform, Bernard et al conducted a teach-ability study that engaged a multi- disciplinary resident cohort group: cardiology, critical care medicine, and anesthesiology. The results of their research revealed all residents, regardless of discipline, made improvements from baseline skill levels when exposed to an electronic learning platform followed by hands- on simulation experience [5].

The Journal of Hospital Medicine published research in 2009 also related to teaching a hospitalist to read basic TTEs. However, the intent was for the hospitalist to then train others following a determination by a cardiologist (that the hospitalist faculty) was a suitable candidate to complete this task [6]. The hospitalist faculty (new instructors) then created didactic curriculum and taught other hospitalists. While significantly different from more typical approaches, it was validated that the hospitalists were able to successfully perform moderate to excellent diagnostic accuracy in 6 unique cardiac pathologies [6]. Critical to note however, is the objective of the research to train hospitalists to identify common TTE pathologies.

In contrast, Tanzola and colleagues [4] conducted research involving a small cohort of 10 medical residents. Following a pretest, the residents were given four presentations on focused transthoracic echocardiography, each three hours in length. This totaled 12 contact hours of TTE content. After the didactic presentations they were administered a posttest to evaluate learning. Despite a comparatively large exposure to the material results showed only modest improvement in posttest scores. Similar findings were published by Martin et al. [7]. Their research aimed to evaluate the effectiveness of a training program for hospitalists with the objective being that the hospitalists could use hand-held devices to identify and create bedside TTE images. The learners were exposed to 35 cardiac echocardiograms and subsequently could identify but not always replicate the basic views. This supports the theory that didactic learning methods alone may not be adequate to effectively improve competency or for attaining knowledge [4].

The consistent theme in the published literature emphasizes two different curriculum and teaching methods. The first is didactic teaching formats, however the length and content of the curriculum was noted to be quite varied. The second curriculum method, a blended method of sorts, reveals a positive relationship between didactic and simulation. These concepts support the blended format used in this study. Our literature search also found recommendations made by the American Society of Echocardiographers related to training focused bedside echocardiography. While significantly more in depth, specific language is found addressing the importance of both didactic and hands on simulation [10].

## Methods

Following university IRB approval, 26 graduate student registered nurse anesthetists (SRNAs- the study participants) all enrolled in an Advanced Health Assessment course that included extensive POCUS curriculum were invited to participate. Participation was voluntary and students were informed that declining participation would not adversely affect their matriculation. After explanation of the research, a written information sheet was distributed to each student, and time was allotted for questions. Study participants were administered a pretest that included a demographic section (gender and age range) and two additional questions about receiving formal training in ultrasound and/or ultrasound technology. If participants had received formal training in ultrasound and/or ultrasound technology, they were asked to explain. We considered that prior exposure to Transthoracic echocardiography (TTE) would alter study results.

In addition, each study participant was asked to generate a 4- number code and place it at the top righthand corner of the pretest. They were instructed to use this same 4- number code for the posttest so scores could be compared individually (in addition to group mean scores). All doctoral students agreed to participate in the study.

The images provided on the pretest included four basic views of common focussed TTEs, converted from sonogram to graphic form: sub-costal 4 chamber, apical 4 chamber, parasternal short axis at the level of the papillary muscles (PSAX), and parasternal long axis (PLAX). These same four views were also presented as actual TTE sonograms, for a total of eight questions. Participants were allowed 20 minutes to complete the eight question pretest. Following the pretest, an existing, formalized, well established, and blended curriculum was provided that included, but not limited to, basic TTE view identification. The curricular program was part of an existing training program created to assist anesthesia providers to learn both basic four-view and focused TTE, but also generalized bedside POCUS skills. The didactic presentation was one hour in length and included only the focused TTE views with descriptions that would be included in the pre and posttest. It should be noted that coupled with the didactic curriculum were three projection screens in the auditorium classroom setting for viewing while listening. Following the didactic curriculum, a second hour of live hands- on simulation practice took place. This involved not only identification of the four views but also production of the views on live models. After the completion of the blended curriculum and exposure to the four basic and focused bedside TTE views, the study participants completed the posttest. The posttest was the same as the pretest in both structure and images. Upon data collection, a t-test scoring was selected and used to complete the analysis.

**Fig. 1:**
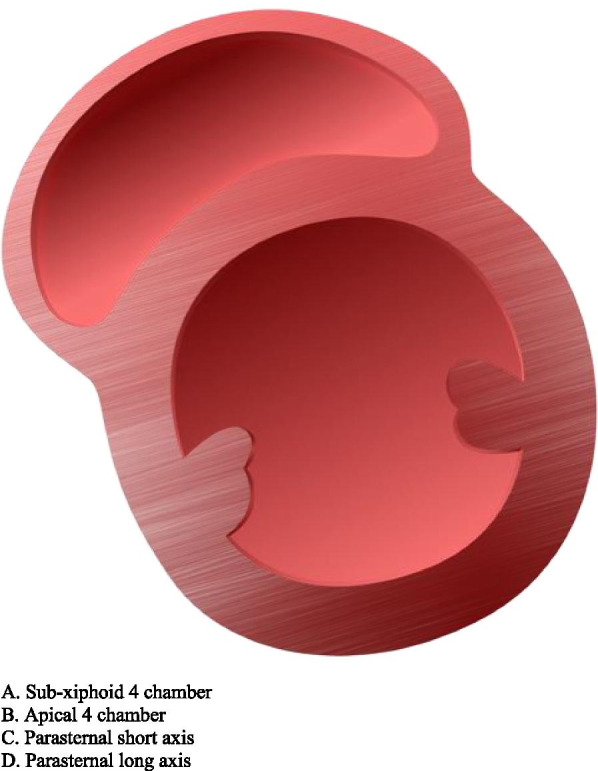
Example of actual graphic study question

**Fig. 2:**
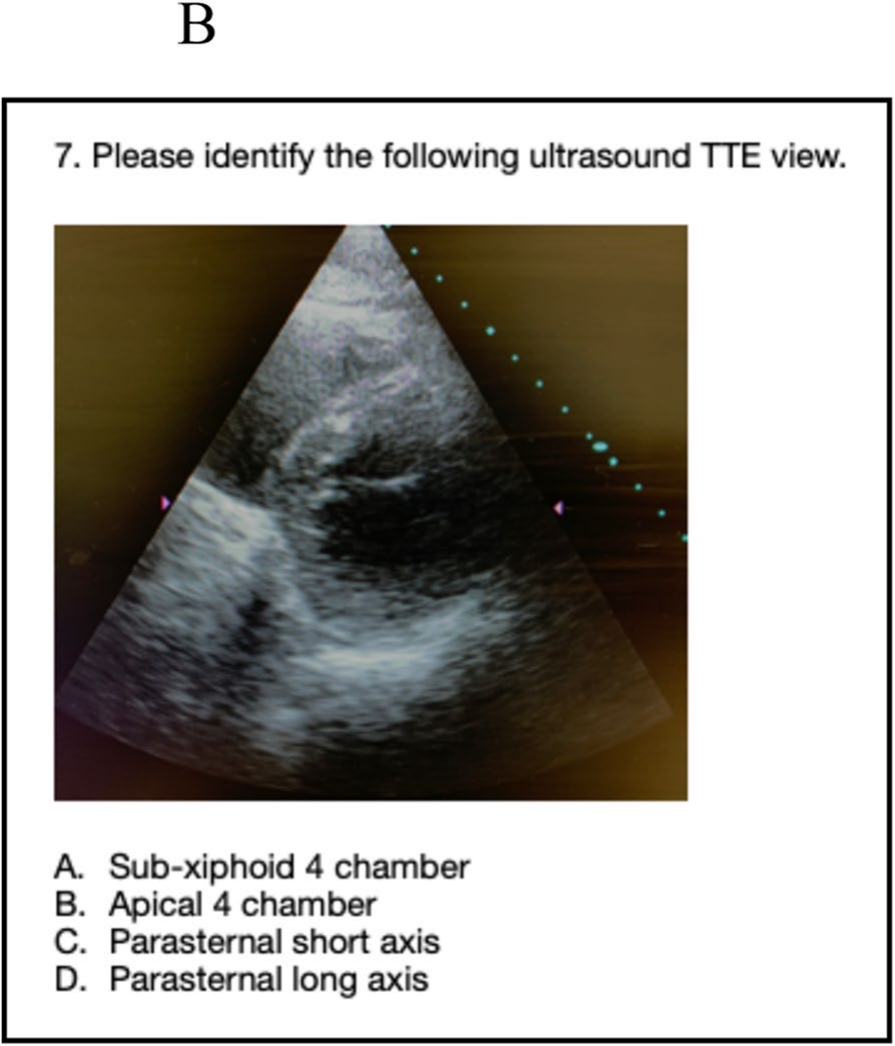
Example of actual sonogram study question

An example of the actual exam is listed below in Figs. 1 and 2. Figure 1 shows a graphic of a PSAX TTE view. Figure 2 shows the corresponding TTE sonogram of the same PSAX view.

**Fig. 3:**
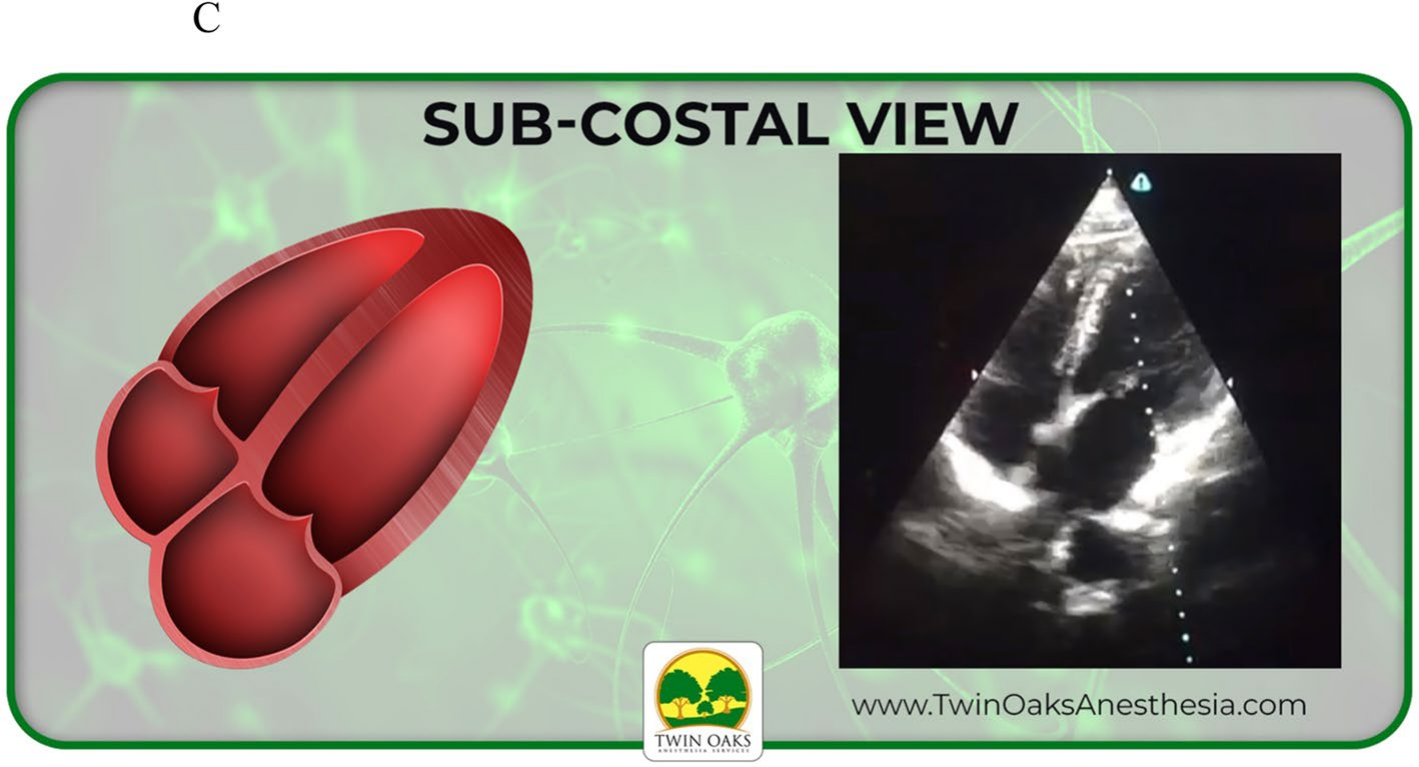
Study graphic and corresponding sonogram of sub-costal TTE view

**Fig. 4:**
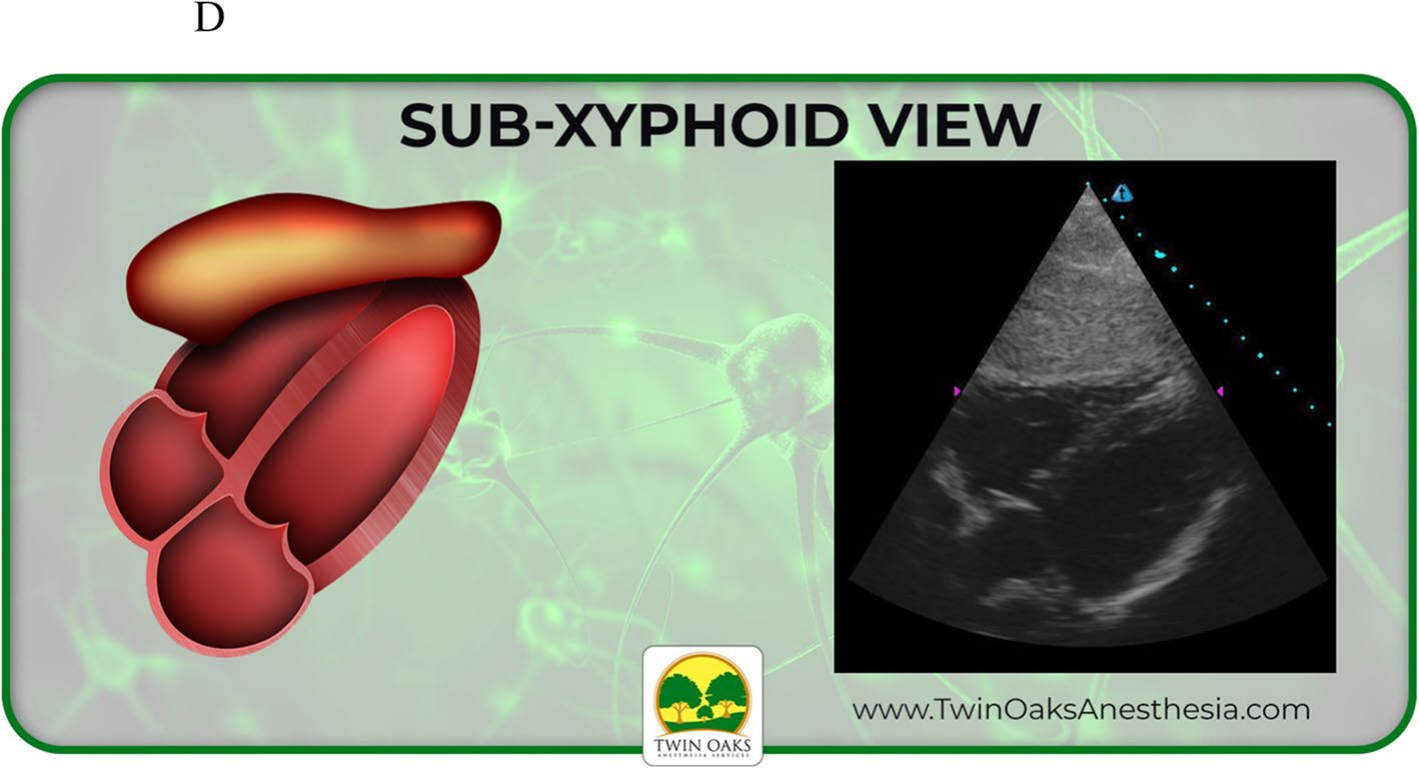
Study graphic and corresponding sonogram of sub-xyphoid TTE view

**Fig. 5:**
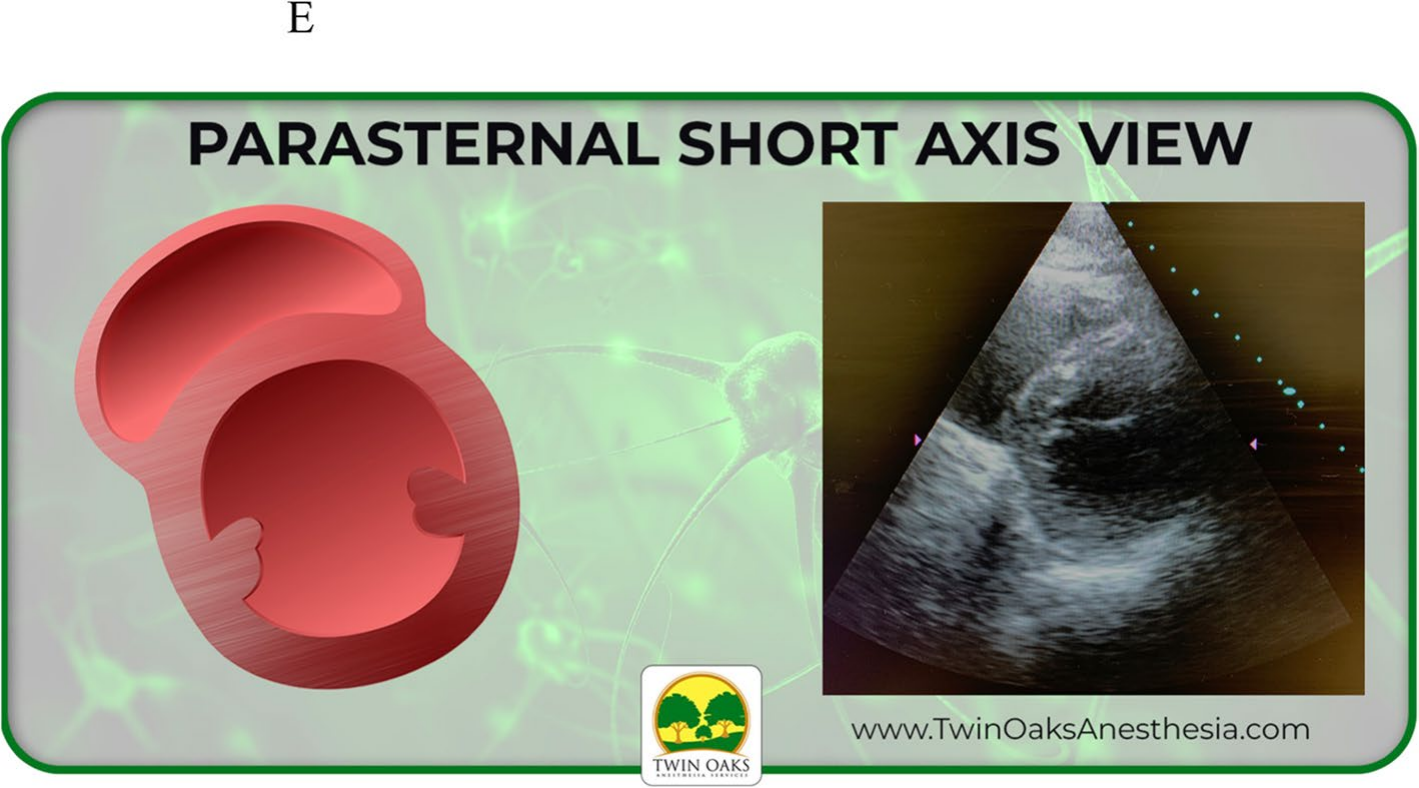
Study graphic of parasternal short axis graphic and corresponding TTE view

**Fig. 6:**
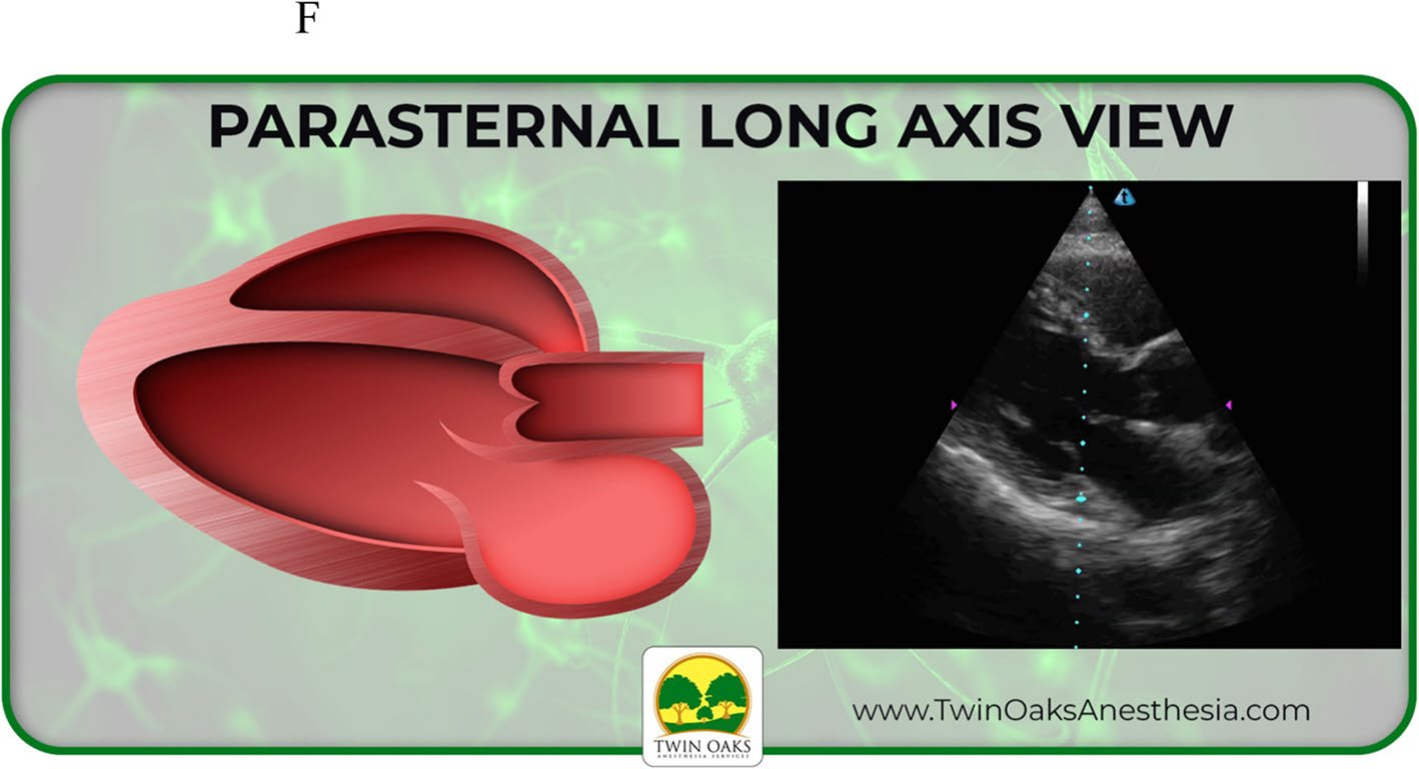
Study graphic of parasternal long axis graphic and corresponding TTE view

See also the learning graphics and corresponding TTE images included in the study. They have been placed into summary form for viewing and include sub-xiphoid 4 chamber, apical 4 chamber, as well as parasternal long (PLAX) and short axis (PSAX). See Figs. 3, 4, 5, and 6 below.

## Results

The average pretest score was 59% and after the blended two hour curriculum, the average posttest scores increased to 95%. A total of four participants (4/26) scored 100% on both the pre and posttest. Notably, 18 of the 26 participants (18/26) scored 100% on the posttest. All but one participant demonstrated a significant increase from pre to posttest scores. A paired t- test was applied to the pretest and posttest scores of the 26 students participating in the study. There was a significant difference in the pretest scores (M = 58.65, SD = 23.66) for interpreting the four most common transthoracic echocardiography (TTE) images compared to the posttest scores (M = 95.19, SD = 9.41) of the same images after the blended curriculum [observed t statistic 7.24, *P* < 0.001; 95% CI [26.15, 46.93]. The application of the t test was justified by Shapiro-Wilk normality test; primarily due to a -12.5 difference between post and pre score. It is worth noting that one participant produced the exact same low score on both pre and posttest. This may be due to an error in data collection, or lack of interest in study participation. When removed from the overall analysis, the results reflect a more favorable post test score. See the below Table 1, for raw collected data from the study.

**Table 1:** Original data collection illustrating pre and post test scores for the study

Study participant number	Pretest scores (%)	Posttest scores (%)	(1) Used ultrasound (2) Formal US training
1	37.5	100	
2	37.5	100	1 (IV insertions)
3	87.5	100	
4	37.5	87.5	
5	62.5	100	
6	100	100	
7	25	100	
8	37.5	87.5	
9	100	87.5	
10	75	87.5	2 (curriculum /regional course)
11	62.5	87.5	
12	50	100	2 (IV and arterial catheter)
13	75	100	
14	50	100	
15	25	100	
16	50	100	1 (IV insertions)
17	62.5	100	
18	25	100	
19	62.5	62.5	
20	50	100	1 (IV insertions)
21	50	75	
22	62.5	100	
23	100	100	
24	50	100	
25	50	100	
26	100	100	

## Discussion

The outcome of this research supports several distinct concepts. First, SRNAs successfully identified the four basic and focused transthoracic echocardiography (TTE) views following an established TTE blended curriculum. The curriculum content is owned by Twin Oaks Anesthesia and developed and delivered by Twin Oaks Anesthesia faculty. Twin Oaks Anesthesia seminars specialize in comprehensive CE approved courses for anesthesia techniques with emphasis on ultrasound guidance for anesthesia professionals and other healthcare related disciplines. Second, it was discovered that the existing content delivery method was efficacious for learning, and teaching methods were more than satisfactory and validated by improved posttest scores. This notion, while not a study question, was considered an incidental finding. This project demonstrated that SRNAs are adept at absorbing and mastering the content, but the way the content was delivered appears highly successful for learning. The blended curriculum for example involved a didactic presentation in a slide show format with real life images from clinical scenarios. After the didactic presentation, there was immediate live model (standardized volunteers) scanning and the learners demonstrated attainment of skills in a controlled and supportive simulation environment. It is noteworthy that all study participants were exposed to a ‘fundamentals of ultrasound’ curriculum the previous day prior to the TTE section. The study faculty believe it is essential for learners to first comprehend ultrasound physics, terminology, and machine function, and these concepts should be taught prior to attempting basic 4 view TTE assessments.

The results illustrate the effectiveness of the program. Note the increase from average pre test scores of 59% to average post test scores of 95%. This project was part of an experimental pilot program to evaluate if incorporating comprehensive POCUS curriculum, including use of an existing basic and focused TTE platform, successfully achieved the objectives of the required advanced health assessment course and as mandated by the Council of Accreditation (COA). The hands- on simulation portion of the blended curriculum was valued and enhanced SRNA learning. This was based on not only posttest scores but also by informal student feedback (in evaluations of the course) and during general conversation following the experiences and program.

This blended curriculum was successful for this cohort of students, and an extensive literature review revealed that variations in methods of teaching may lead to inconsistencies in learner outcomes. Several studies deemed a blended type teaching platform led to successes in skill attainment and conceivably, a non- blended curriculum or simply didactic curriculum may be a barrier to the successful attainment of skills for any healthcare provider. For example, Coker and Zimmerman (2017) defined and emphasized the importance of bedside focused transthoracic echocardiography (TTE) skills for the anesthesia community to embrace. They defined basic TTE skills as understanding and creating four views: a sub-costal 4 chamber view; apical 4 chamber view; parasternal short axis at the level of the papillary muscles (PSAX); and parasternal long axis [1]. The value of attaining these basic TTE skills is not in dispute, however the teaching method used may create inconsistencies in learning. Interesting to note that a blended teaching platform appears to support attainment of skills greater than lone didactic formats.

### Limitations

We recognize several limitations to the study. A co-investigator was also the primary curriculum presenter. This could potentially influence bias in the results. Another limitation to note is the duplicity in the pretest and the posttest as the images used were the same for both. The argument could be made that simple patten recognition is possible of nearly any reasonable study participant, and that testing should have included a posttest highlighting the same content but ‘different’ images. While we foresaw this as a possible weakness, the opposite can also hold true. True data collection, demonstrating patten recognition can be determined by administering the exact same pre and posttest. This would validate the rationale for employing the same tests to study participants. We also recognize that the testing design, while effective is a limitation of the data collection. The use of multiple choice design can introduce some bias as there is a generally accepted notion that study participants have essentially a 1 in 4 chance of guessing the correct answer. This however is offset by the standardization power, and convenience of rapid data collection. Another limitation is the population size. We were fortunate to have a robust (100% of possible 26 participants) study population, but it consisted of a relatively low number. We hope that future studies will exceed our capabilities if the study is repeated. We also acknowledge that while 18 of the 26 study participants scored 100 on the post test, there were 4 who also scored a 100 on the pretest.

The use of immediate simulation hands-on practice also may have played a role in SRNA engagement, understanding and retention, thereby impacting their overall increase in posttest scores. Searching the literature confirmed that excluding the hands- on simulation component lowered posttest scores compared to when a hands-on component was included. This also gave us rationale for our study design. While not a typical pretest- posttest research design, we trust that immediate hands- on simulation training holds great value to not only assist SRNAs in absorbing the content, but also enhances clinical relevance.

## Conclusion

The results of our data collection revealed two distinct concepts. Following a blended curriculum that included a didactic component and a one- hour live, hands- on simulation, posttest scores significantly increased compared to pretest scores. And the existing transthoracic echocardiography (TTE) blended curriculum developed and produced by the Twin Oaks Anesthesia is high efficacious for learning basic, focused 4 view TTE. More research is needed specific to POCUS, focused TTE, and SRNA learning.

## Data Availability

All raw data are readily available per request. and authors contributions and acknowledgements are included in the manuscript.

## References

[1] Coker B, Zimmerman J (2017). Why anesthesiologists must incorporate focused cardiac ultrasound into daily practice. Anesth Analg.

[2] Neelankavil J, Howard-Quijano K, Hsieh TC, Ramsingh D, Scovotti JC, Chua JH, Ho JK, Mahajan A (2012). Transthoracic echocardiography simulation is an efficient method to train anesthesiologists in basic transthoracic echocardiography skills. Anesth Analg.

[3] Kusunose K, Yamada H, Suzukawa R, et al. Effects of transthoracic echocardiographic simulator training on performance and satisfaction in medical students. J Am Soc Echocardiogr. 2016. 10.1016/j.echo.2015.12.002 26743736

[4] Tanzola RC, Walsh S, Hopman WM (2013). Brief report: focused transthoracic echocardiography training in a cohort of Canadian anesthesiology residents: a pilot study. J Can Anesth.

[5] Bernard, A. Pascale C, Dion F. et al. abc Arch Cardiovasc Dis. 2019;112(10):576-584 10.1016/j.acvd.2019.06.001 31350012

[6] Lucas B, Candotti C, Margeta B (2009). Diagnostic accuracy of hospitalist-performed hand-carried ultrasound echocardiography after a brief training program. J Hosp Med.

[7] Martin LD, Howell EE, Ziegelstein RC, Martire C, Shapiro EP, Hellmann DB (2007). Hospitalist performance of cardiac hand-carried ultrasound after focused training. Am J Med.

[8] Vignon P, Dugard A, Abraham J, Belcour D, Gondran G, Pepino F (2007). Focused training for goal-oriented hand-held echocardiography per- formed by noncardiologist residents in the intensive care unit. Intensive Care Med.

[9] Gibbs V (2015). The role of ultrasound simulators in education: an investigation into sonography student experiences and clinical mentor perceptions. Ultrasound..

[10] Spencer K, Kimura B, Korcarz C (2013). Focused cardiac ultrasound: recommendations from the American Society of Echocardiography. J Am Soc Echocardiogr.

